# Transconjunctival excision of an orbital conjunctival cyst using computer‐assisted 3‐D surgical planning in a dog

**DOI:** 10.1002/ccr3.4345

**Published:** 2021-07-06

**Authors:** Jessica B. Burn, András M. Komáromy, Dodd G. Sledge, Rebecca Smedley, Sarah E. Coe, Sun Young Kim

**Affiliations:** ^1^ Department of Small Animal Clinical Sciences College of Veterinary Medicine Michigan State University East Lansing MI USA; ^2^ Diagnostic Center for Population and Animal Health Michigan State University Lansing MI USA; ^3^ Department of Veterinary Clinical Sciences College of Veterinary Medicine Purdue University West Lafayette IN USA

## Abstract

Investigation of exophthalmos and blood‐colored discharge from the left ventral punctum in a dog was consistent with a conjunctival cyst in the orbit. 3‐D prints of the cyst and surrounding facial bones identified a successful transconjunctival approach without an orbitotomy and patency of the left lacrimal duct was reestablished.

## Graphical abstract

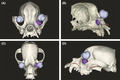

## 1 INTRODUCTION

Investigation of exophthalmos and blood‐colored discharge from the left ventral punctum in a dog was consistent with a conjunctival cyst in the orbit. 3‐D prints of the cyst and surrounding facial bones identified a successful transconjunctival approach without an orbitotomy, and patency of the left lacrimal duct was re‐established.

Conjunctival cysts in dogs originate from either the conjunctival epithelium or the epithelium of the lacrimal duct are frequently reported in the orbit in dogs.^1^, ^2^, ^3^ They may be of primary or secondary origin,^4^ and complete surgical excision is typically curative. The underlying mechanism of cyst formation remains unclear; however, congenital duct malformation, production of abnormal secretory material, trauma, and/or inflammation leading to lacrimal gland hypersecretion and subsequent injury to the walls of the ducts are speculated to lead to passive dilation and cyst formation.^5^, ^6^, ^7^

Successful medical and/or surgical treatments for conjunctival cysts include injection of a sclerosing agent such as 1% polidocanol, surgical marsupialization, and orbitotomy with enucleation to access and surgically excise the cyst.^3^, ^8^, ^9^, ^10^, ^11^ Treatment choice is dependent on the size and location of the cyst and the involvement of adjacent anatomical structures. Surgical approaches are often complicated by the presence of the globe itself, the proximity of nerves and large vessels, and the need to work in a small space surrounded by bones and ligaments. This report details the surgical planning and transconjunctival excision of a conjunctival cyst in a dog using a patient‐specific 3‐D model.

## 2 CASE HISTORY

A 5‐year‐old neutered male Cavalier King Charles Spaniel dog presented to the Ophthalmology Service of the College of Veterinary Medicine at Michigan State University for evaluation of dark, blood‐colored discharge emanating from the left ventral punctum of 8‐month duration. Treatment for suspected bilateral allergic conjunctivitis was initiated by the referring veterinarian with neomycin polymyxin B dexamethasone 0.1% ophthalmic suspension (USP: Bausch and Lomb) twice daily in both eyes for 2 weeks. The dog's right eye responded well, but the left eye remained abnormal.

## 3 INITIAL CLINICAL FINDINGS

**FIGURE 1:**
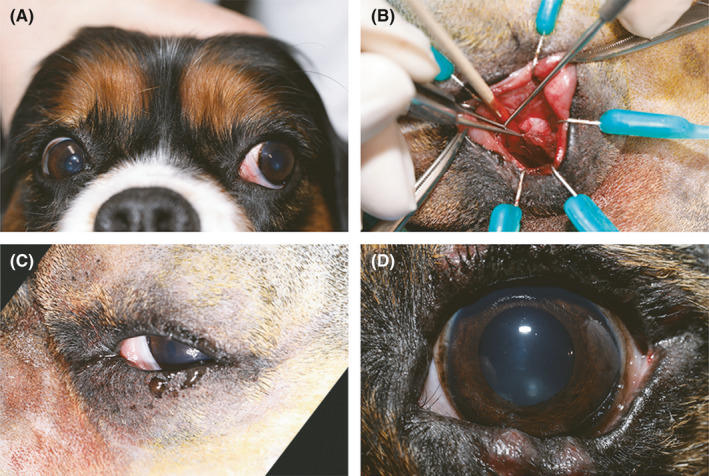
En face view A, of raised left nictitating membrane, exophthalmos, and lateral deviation of the patient's left globe on initial presentation. Mild blood‐tinged discharge is present. Intraoperative photograph B, demonstrating an intact cyst capsule located ventral to the nictitating membrane. Immediate postoperative photograph C, of the left eye demonstrating no major cosmetic changes to the face. Photograph of the left eye D, 2 weeks postoperative. Mild irritation from the temporary tarsorrhaphy was observed along the superior and inferior eyelid margins

Ophthalmic examination yielded a slightly raised left nictitating membrane, mild exophthalmos, and lateral deviation of the left globe. Retropulsion of the left globe yielded a mild stream of thick, dark red discharge and fresh blood from the left lower punctum (Figure 1A) Fluorescein passage tests were negative for both nasolacrimal ducts. Normal airflow was present in both nostrils. The remainder of the ophthalmic and physical examination was unremarkable.

## 4 ANCILLARY DIAGNOSTIC TESTS

**FIGURE 2:**
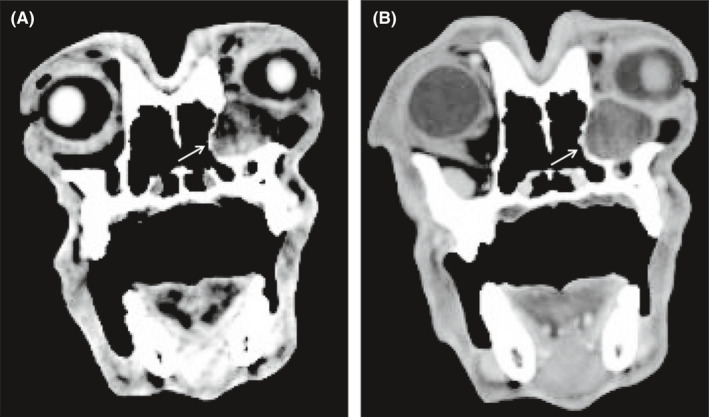
Transverse CT scan in a soft tissue window A, and bone window B, of the skull demonstrating the presence of a cyst ventromedial to the left globe, with adjacent lysis of the orbital bone (arrow)

Computed tomography (CT) of the head revealed a 2‐cm‐diameter, round, well‐circumscribed, thin‐walled, fluid‐filled structure ventromedial to the left globe causing moderate exophthalmos with focal medial displacement and lysis of the osseous orbital wall (Figure 2). Contrast dacrocystorhinography (Omnipaque; iohexol injection 52%; Marconi Medical Systems) of both nasolacrimal ducts revealed normal dye passage, and pooling of dye was observed in the left nasolacrimal duct along the dorsal margin of the mass. Ultrasound‐guided fine‐needle aspiration yielded hemorrhagic fluid with cytologic diagnosis of neutrophilic and macrophagic inflammation and no microorganisms. Aerobic and anaerobic cultures of the cyst contents were negative. A presumptive diagnosis of a nasolacrimal cyst was established. Other differential diagnoses that were considered included neoplasia, foreign body, or granuloma causing obstruction of the nasolacrimal duct.

## 5 SURGICAL MANAGEMENT

**FIGURE 3:**
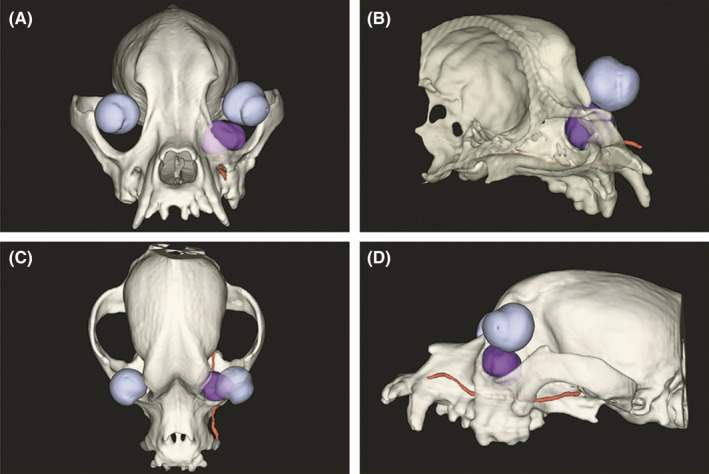
En face A, right oblique B, dorsal C, and left lateral D, views of a digital 3‐D model of the cystic lesion (dark purple) and its surrounding structures including the globe, bony structures, and the infraorbital artery were segmented, and a partial orbitotomy was simulated to expose the cyst using 3‐D modeling software (3D Slicer 4.10.2, slicr.org)

A digital 3‐D model of the cystic lesion and its surrounding structures including the globe, bony structures, and the infraorbital artery was segmented, and a partial orbitotomy was simulated to expose the cyst using 3‐D modeling software (3D Slicer 4.10.2, slicr.org; Figure 3). The cystic lesion and a portion of skull around the lesion, including the zygomatic, lacrimal, and maxillary bones and their associated foramina, were separately 3‐D printed with a 300 μm layer thickness (3‐D Printer: Form 2 Formlabs). A simulated surgery using the 3‐D digital model and manipulation of the 3‐D prints established that a transconjunctival approach to the cyst was feasible without an orbitotomy. The 3‐D prints were gas sterilized with ethylene oxide for use in surgery.

The dog was premedicated (methadone hydrochloride [Methadone: Bioniche Pharma] 0.5 mg/kg IV), induced (alfaxalone [Alfaxan^®^, Jurox Animal Health] 1 mg/kg IV to effect), intubated, and maintained on isoflurane gas (Isoflurane: Isothesia, Henry Schein) in oxygen. Maintenance fluids (Lactated Ringer's Injection Rx, USP: Hospira; 5 mL/kg/hr IV) were administered under anesthesia. The left nictitating membrane was protruded, and an 8‐mm conjunctival incision was made on its palpebral side, approximately 10 mm ventral to the leading edge. The cystic lesion was isolated by surgical dissection, avoiding the angularis oculi vein dorsomedial to the incision, and the deep facial vein, malar artery, infraorbital and maxillary branches of the trigeminal nerve, and the buccal branch of the facial nerve ventromedial to the incision. Gentle traction was applied to exteriorize the cyst for further dissection (Figure 1B). A thickened, fibrous band adhered the medial aspect of the cyst capsule to the ventral aspect of the lytic orbital bone; it was transected to allow for removal of the cyst capsule without fragmentation of the bone. The subcutaneous and conjunctival tissues were closed in a simple continuous suture pattern using 6‐0 polyglactin 910 (Vicryl: Ethicon). A complete temporary tarsorrhaphy was placed using IV tubing for stents and 4‐0 monofilament nylon (Ethilon: Ethicon) to protect the ocular surface. An Elizabethan collar was fitted, and the dog was discharged with oral carprofen (Rimadyl: Pfizer Animal Health) 2.2 mg/kg twice daily and amoxicillin trihydrate/clavulanate potassium (Clavamox: Pfizer Animal Health) 14.5 mg/kg twice daily for 2 weeks.

## 6 HISTOPATHOLOGY

**FIGURE 4:**
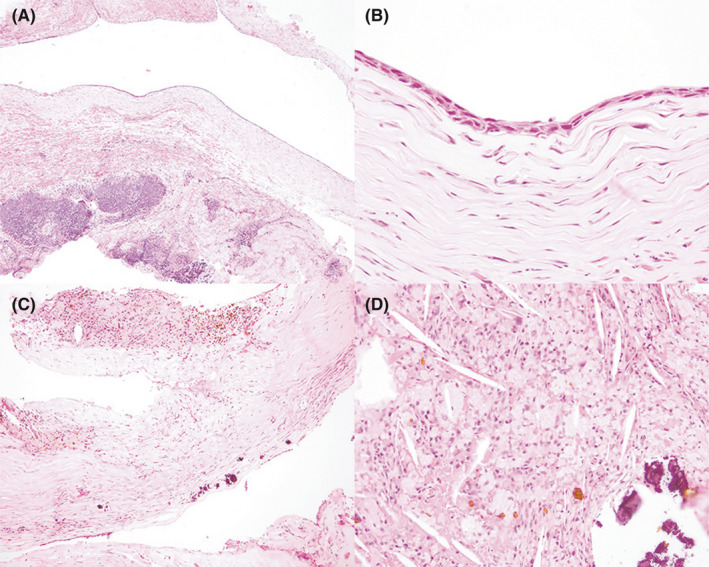
Photomicrographs of an excised conjunctival cyst from a dog A, demonstrating a central cyst lumen lined by attenuated epithelium, supported by a thick band of dense fibrous connective tissue containing deep dense perivascular lymphonodular aggregates. The epithelium lining the cyst wall consists of attenuated stratified squamous epithelial cells B. In segmental regions C, the cyst epithelium is lost, and the exposed stroma contains foci of mineralized debris and regions of chronic hemorrhage infiltrated by hemosiderin‐laden macrophages. The cyst lumen D, contains dense aggregates of foamy histiocytes that often contain intracytoplasmic hemosiderin, extravasated erythrocytes, acicular clefts, and foci of basophilic mineralized debris. Hematoxylin and eosin stain

Histopathologic evaluation of the excised lesion confirmed a benign conjunctival cyst lined by variably eroded to attenuated squamous epithelium with a thick surrounding band of dense collagenous stroma containing occasional perivascular lymphonodular aggregates (Figure 4). Within the cyst lumen and focally within the cyst wall, there was evidence of chronic hemorrhage, including pools of extravasated erythrocytes, aggregates of hemosiderin‐laden macrophages, and aggregates of mineralized debris. The pathologic diagnosis was simple epithelial cyst, which is consistent with the presumptive diagnosis of nasolacrimal cyst previously mentioned.

## 7 OUTCOME

The punctal discharge resolved immediately after surgery (Figure 1C). The temporary tarsorrhaphy sutures were removed at recheck examination 2 weeks postoperatively; a cosmetic outcome was observed (Figure 1D). The left nasolacrimal duct was patent (positive Jones test), retropulsion of the globe no longer resulted in extrusion of discharge from the ventral punctum, and no complications were noted. Follow‐up by telephone conversation 11 months postoperatively confirmed the dog remained asymptomatic with no recurrence of punctal discharge.

## 8 DISCUSSION

The surgical technique and 3‐D modeling described herein allowed for a minimally invasive procedure that eliminated the need to sacrifice the globe or to perform an orbitotomy to access the cyst. To the authors' knowledge, only one other report has documented the use of a patient‐specific 3‐D model to aid in planning the surgical approach to a nasolacrimal duct obstruction in a dog.^12^

Although advanced imaging including dacryocystorhinography and 3‐D CT imaging are valuable tools to identify orbital lesions and their surrounding structures and to develop a treatment plan,^13^, ^14^, ^15^, ^16^ these imaging modalities only provide a digital rendition that cannot be handled or manipulated by the surgeon intraoperatively. In contrast to rendered images, segmented 3‐D surface models in this report allowed for digital manipulation of individual anatomical structures with an unlimited number of trials.^12^, ^17^ The 3‐D printed portion of the skull and of the cyst allowed for preoperative measurement of the cyst and identification of the best surgical approach. Although the virtual surgery indicated a partial orbitotomy would optimize exposure of the lesion, the tangible 3‐D printed models showed the possibility of excision of the cyst with gentle traction via a transconjunctival approach. The 3‐D model provided sufficient anatomical detail to explore and remove the cyst while avoiding the deep facial vein, anastomotic branch to the ventral external ophthalmic vein, malar artery, zygomaticofacial nerve, and zygomaticotemporal nerve to minimize the risk of hemorrhage and to preserve normal motor and sensory function. With the degree of lysis present, there was concern for bone fragmentation during removal of the embedded cystic tissue. Therefore, as much of the exposed cystic material as possible was debrided for biopsy, and a portion of a fibrotic band that was attached to the cystic capsule was transected and left adhered to the bone. Histopathological results confirmed a benign cyst; as such, no revision surgery was performed to extract the remaining fibrous tissue.

Intralesional treatment with a sclerosing agent such as 1% polidocanol could have been considered for this case (Zimmerman *et al*, 2019); however, the owners elected for surgical excision to maximize the potential for resolution and minimizing the risk of nasolacrimal duct obstruction and blood‐colored discharge. Neoplastic diseases, such as nasal adenocarcinoma, osteosarcoma, lymphoma, and meningioma, were considered as differential diagnoses prior to sampling.^18^ In contrast to the marginal excision required for removal of a benign cyst, as was described in this case, a malignant lesion would necessitate orbitotomy or exenteration in an attempt to achieve clean margins.

Streamlining the surgical approach to preserve the globe and bony structures surrounding the globe and only printing the portion of the skull of interest minimized surgical time and the associated anesthetic and surgical costs.^12^ A printing time of only 1.5 hours and material costs of approximately $10 USD were incurred in this report.

This case report highlights the benefits of CT imaging in concurrence with 3‐D surface modeling and printing to facilitate planning a minimally invasive transconjunctival approach to successfully remove a conjunctival cyst, while sparing the globe and other orbital structures. Our surgical outcome was excellent with normal function of the nasolacrimal duct and no postoperative complications.

## CONFLICT OF INTEREST

None declared.

## AUTHOR CONTRIBUTIONS

JBB: Involvement in the clinical case, manuscript preparation and revision, and compilation of figures. AMK: Involvement in the clinical case, supportive role in manuscript preparation and revision, and formatting of figures. DGS: Contribution to the histopathology section of the manuscript and provision of a histopathology figure. RS and SEC: Involved in histopathology preparation and analysis. SYK: Involvement in the clinical case, supportive role in manuscript preparation and revision, provision of CT images, and provision of the 3‐D printed model.

## ETHICAL APPROVAL

This study was carried out in compliance with the ARVO Statement for the Use of Animals in Ophthalmic and Vision Research and Michigan State University Institutional Animal Care and Use Committee.

## CONSENT STATEMENT

Published with written consent of the patient.

## Data Availability

Data sharing not applicable to this article as no datasets were generated or analyzed during the current study.
